# Associations between common respiratory viruses and invasive group A streptococcal infection: A time‐series analysis

**DOI:** 10.1111/irv.12658

**Published:** 2019-06-25

**Authors:** Brechje de Gier, Bart J. M. Vlaminckx, Sjoukje H. S. Woudt, Nina M. van Sorge, Liselotte van Asten

**Affiliations:** ^1^ Center for Epidemiology and Surveillance of Infectious Diseases National Institute for Public Health and the Environment Bilthoven the Netherlands; ^2^ Medical Microbiology and Immunology St Antonius Hospital Nieuwegein the Netherlands; ^3^ Medical Microbiology University Medical Center Utrecht, Utrecht University Utrecht The Netherlands

**Keywords:** influenza, periodicity, respiratory tract infections, statistical models, *Streptococcus pyogenes*

## Abstract

**Background:**

Invasive infections by group A S*treptococcus* (iGAS, *Streptococcus pyogenes*) have a winter seasonality which largely coincides with the season for influenza and other respiratory viruses. Influenza superinfections with GAS have been described to occur regularly and to show a severe clinical picture with high mortality. We aimed to study the extent to which influenza A and B viruses (IAV and IBV), respiratory syncytial virus (RSV) and rhinovirus circulation contribute to iGAS incidence and severity.

**Methods:**

Time‐series regression models were built to explore the temporal associations between weekly laboratory counts of IAV, IBV, RSV and rhinovirus as independent variables and weekly counts of GAS disease notifications or laboratory GAS cultures as dependent variables.

**Results:**

The weekly number of IAV detections showed a significant temporal association with the number of notifications of streptococcal toxic shock syndrome (STSS), a severe complication of iGAS. Depending on the season, up to 40% of all notified STSS cases was attributable to IAV circulation. Besides STSS, none of the other iGAS manifestations were associated with a respiratory virus.

**Conclusions:**

Our study found an ecological temporal association between IAV and STSS, the most severe complication of iGAS. Future studies are needed to confirm this association and assess the possible preventability of STSS by influenza vaccination, especially in the age group 60 years and older.

## 1 INTRODUCTION

*Streptococcus pyogenes* (group A *Streptococcus*, GAS) can be carried harmlessly in the respiratory tract and on the skin, but also causes a wide range of non‐invasive (impetigo, pharyngitis) and invasive (necrotising fasciitis, pneumonia, sepsis) diseases. As a complication of invasive GAS infection (iGAS), streptococcal toxic shock syndrome (STSS) may develop, which has a mortality rate of around 40%.^1^, ^2^ Known risk factors for severe iGAS include diabetes and older age.^3^ iGAS disease occurrence has a strong seasonality in temperate climates, with higher incidences in winter and early spring, largely coinciding with the season for influenza and other respiratory infections.^3^ The drivers of iGAS seasonality are thus far unexplained, but GAS superinfections on common respiratory viruses such as influenza have been reported.^4^, ^5^ Notably, during the 1918 influenza pandemic, streptococcal superinfections were important causes of death, which besides *Streptococcus pneumoniae* also included GAS.^6^

In 2010‐2011, an increased iGAS incidence was observed in the United Kingdom during a high influenza season.^7^ Record linkage with influenza surveillance data showed that 9% of iGAS cases also had a laboratory‐confirmed influenza infection, which is likely to be an underestimation of the true coinfection rate as testing for influenza infection is not common. Case series of influenza‐iGAS co‐infections have shown a severe clinical picture with mortality rates around 70% but are based on low numbers.^8^, ^9^

Therefore, the extent to which influenza contributes to iGAS seasonality or severity remains unknown. Importantly, in the case of such an association, a proportion of iGAS cases is potentially avertable by influenza prevention. Patients with iGAS disease are not usually tested for influenza infection, which complicates investigation of a link between the two infections. However, comprehensive surveillance systems both of respiratory pathogens and of iGAS disease separately offer an opportunity to explore the association between iGAS and respiratory pathogens through modelling of their time series. In the absence of individual‐level data on co‐infections, such time‐series analyses using population‐level data are an important tool for hypothesis generation and directing future in‐depth research. While time series have been used to estimate the association between respiratory virus infections and infections with another *Streptococcus* species (*Streptococcus pneumoniae*),^10^ such population‐level studies with GAS infections are very scarce. One such study, by Allard et al,^11^ reported a specific association between influenza B virus and GAS laboratory detections in Montréal, Canada.

Our research question was whether influenza (A or B) seasonality was associated with iGAS disease in the Netherlands, and if so, whether this association was specific to influenza or whether it existed for any respiratory virus with a winter season. We constructed time‐series regression models to estimate the temporal, population‐level associations between weekly respiratory virus counts (influenza A and B, RSV and rhinovirus) and weekly counts of iGAS disease notifications or microbiological GAS cultures.

## 2 METHODS

### 2.1 Data sources

#### 2.1.1 Respiratory virus detections

Weekly numbers of respiratory virus detections from week 1 2008 to week 26 2018 were extracted from the Virological Weekly Reports (VWR), a voluntary and sentinel laboratory surveillance system established by the Dutch Working Group on Clinical Virology. Participating laboratories report the weekly counts of detections for 46 pathogens. No patient‐level data are collected. Participation has been consistent over the study period, with a median of 20 (range 17‐21) laboratories reporting weekly, and the data were shown to be representative of epidemiological trends on the national level in a recent evaluation comparing the VWR to mandatory notification data.^12^ The VWR participating laboratories include smaller and larger laboratories plus several academic hospitals. A study conducted in 1999‐2000 estimated the national population coverage of the VWR at 73% for influenza, with 17 participating laboratories at the time.^13^ For the current study, detections of common respiratory viruses (influenza A virus (IAV), influenza B virus (IBV), respiratory syncytial virus (RSV) and rhinovirus) were included.

#### 2.1.2 iGAS disease notifications

Notifications of iGAS disease per week (week 1 2011‐week 26 2018) were extracted from the national database for notifiable diseases “OSIRIS.” In the Netherlands, three manifestations of iGAS disease are notifiable since 2011: STSS, necrotising fasciitis and puerperal sepsis. Since June 2016, also puerperal (ie, within 3 weeks post‐partum) fever with a GAS culture from a non‐sterile site (ie, culture from the female urogenital tract) is notifiable. Puerperal sepsis or puerperal fever will henceforth be referred to as puerperal GAS. STSS and necrotising fasciitis are notifiable if GAS is cultured from a normally sterile site, or when GAS is cultured from a normally non‐sterile site, in the absence of another micro‐organism that can cause the disease. Since notifiable by law, notifications of iGAS diseases STSS, necrotising fasciitis and puerperal GAS should include all cases occurring throughout the Netherlands, some underreporting notwithstanding.

#### 2.1.3 GAS cultures

Weekly numbers of GAS‐positive cultures (week 1 2008‐week 52 2017), by specimen type, were derived from the national Infectious Diseases Surveillance Information System—Antimicrobial Resistance (ISIS‐AR).^14^ This surveillance system is a combined initiative of the Dutch Ministry of Health, Welfare and Sport and the Netherlands Society for Medical Microbiology, and is maintained by the Centre for Infectious Disease Control at the National Institute for Public Health and the Environment and the participating medical microbiology laboratories (MMLs). All results of antimicrobial susceptibility testing (AST) from participating laboratories are uploaded in this surveillance system. As AST is routinely performed for all GAS isolates, we assumed counts of GAS isolates to be representative for the number of GAS diagnoses. However, to avoid overestimation of diagnoses by including follow‐up cultures of the same disease episode, we included only isolates that were not preceded by a GAS isolate from the same patient in the 6 months before. Coverage of ISIS‐AR has increased during the study period (2008‐2017) from around 50% to 70% of the number of laboratories in the Netherlands; however, the exact catchment areas of the laboratories are unknown. Isolates from blood, upper and lower respiratory tract material, genital tract material and pus or wounds were separately included in the analysis. The representativeness of the GAS culture data for GAS disease will vary per specimen type: culturing upper respiratory tract specimens for, for example, tonsillitis is likely to occur regularly, but is not a standard procedure. On the other hand, blood cultures are always performed in sepsis cases.

### 2.2 Statistical models

We used time‐series regression to estimate the temporal association between respiratory virus detections and both iGAS notifications and GAS cultures. Negative binomial models were constructed with weekly counts of respiratory viruses as independent variables and weekly counts of iGAS disease notifications (STSS, necrotising fasciitis or puerperal GAS) or GAS cultures (per specimen type) as dependent variables. Building a separate model per each dependent variable, we thus built eight models. For estimating the association between the weekly counts of respiratory virus detections and iGAS disease notifications, the period of week 1 2011 to week 26 2018 was included. The GAS culture data were available from week 1 2008 up to week 52 2017; therefore, this period was included in the GAS culture models. For ease of interpretation, we used an identity link, which assumes the effects of different respiratory viruses on GAS incidence to be additive rather than multiplicative. In model selection steps, *P*‐values were used to determine whether a parameter was associated with the dependent variable. Parameters with *P*‐values <0.1 were included in a stepwise model selection.

To allow a baseline seasonal variation in GAS, independent of respiratory virus activity, first a sine wave was modelled before including the respiratory virus variables. For each GAS outcome, initially a model was constructed with four sets of sine/cosine terms, and the number of significant sine/cosine pairs was determined by backward selection. Linear time trends were added only when significant. For all four respiratory viruses, we also explored whether they were significantly associated with GAS when a time lag was taken into account (ie, whether a current increase in virus detections was predictive of current GAS or GAS in later weeks). Time lags of zero up to 4 weeks were explored in this manner. Negative (inverse) associations between respiratory viruses and GAS were disregarded, as we assumed a protective effect of respiratory viruses on GAS disease to be biologically implausible.

If the number of IAV detections showed an overall significant association with GAS, we explored whether a time‐dependent approach improved the model fit, that is separating IAV detections into separate variables per season (defined as week 27‐week 26 the next year). This was done because IAV strain dominance and antigenic drift are known to result in season‐specific influenza epidemiology (eg, severity and attack rate differ from season to season).^15^

To estimate the fraction of iGAS disease attributable to respiratory viruses, beta coefficients from the final model were multiplied by the observed count of virus detections. Analyses were performed in Stata version 15 (StataCorp).

## 3 RESULTS

**Figure 1:**
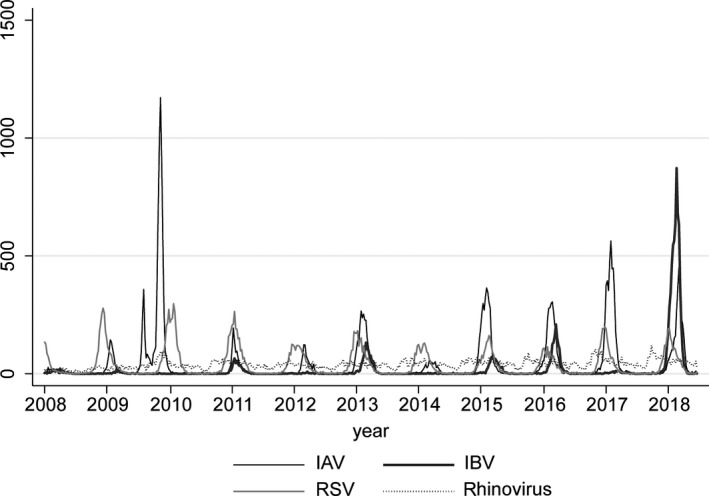
Weekly number of detections of IAV, IBV, RSV and rhinovirus in the Virological Weekly Reports, week 1 (January) 2008 ‐week 26 (June) 2018

**Figure 2:**
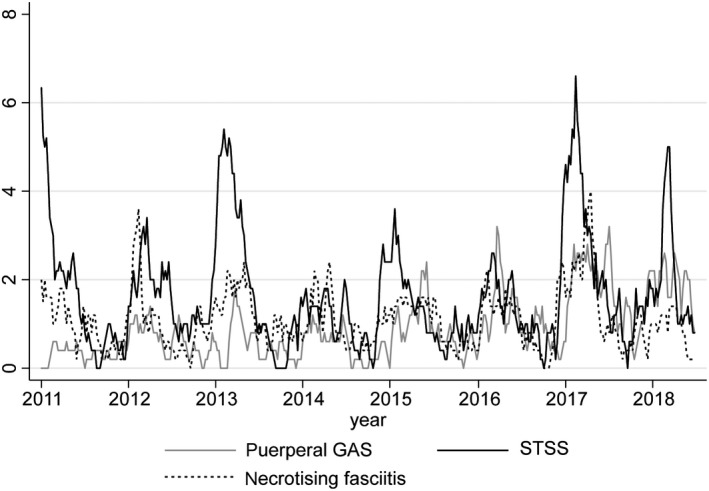
Five‐week moving average of the number of notifications of STSS, necrotising fasciitis and puerperal GAS, week 1 (January) 2011 ‐week 26 (June) 2018

**Figure 3:**
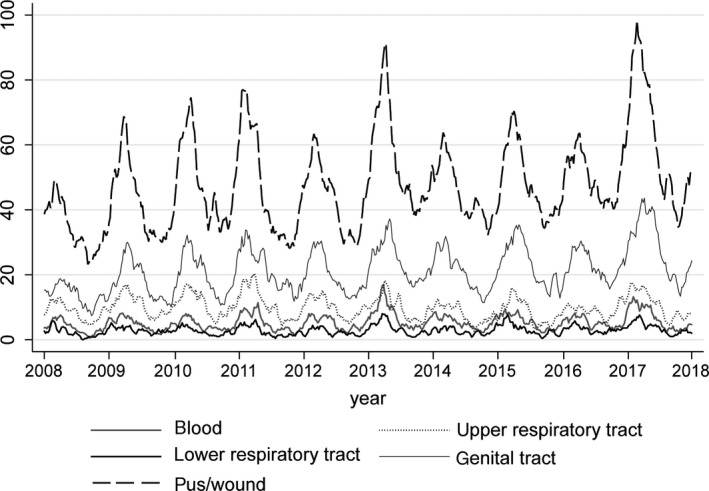
Five‐week moving average of the number of GAS‐positive cultures in ISIS‐AR, by specimen type, week 1 (January) 2011 ‐week 52 (December) 2017

Weekly numbers of detections of respiratory viruses between January 2008 and June 2018 reflected the typical seasonality of respiratory viruses IAV, IBV and RSV, that is with clear annual peaks around winter and almost absent outside of their season (Figure 1). Rhinovirus detections remained quite consistently present throughout the year. All GAS disease notifications and cultures also showed marked seasonality (Figures 2, 3), but retained a baseline presence throughout the year. Annual totals of respiratory virus detections, iGAS disease notifications and GAS isolates are shown in Table S1.

### 3.1 Time‐series regression

In all models, one set of sine/cosine terms (of 1‐year periodicity) showed the best fit. A secular time trend improved the fit of (and was therefore included in) all models with GAS culture numbers as dependent variables, likely reflecting the increasing number of laboratories contributing to the data over the study period. Detections of respiratory viruses IAV, IBV, RSV and rhinovirus were not significantly associated with counts of any of the GAS culture specimen types, or notifications of necrotising fasciitis or puerperal GAS, either in the same week or with any of the four explored time lags.

**Figure 4:**
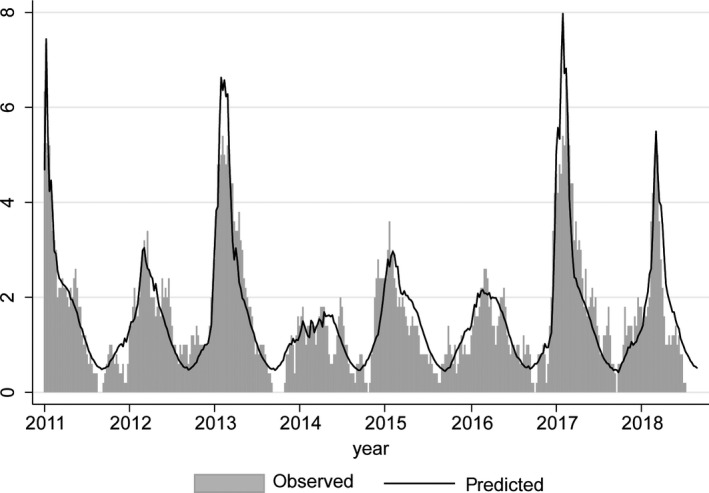
Five‐week moving average of the weekly number of STSS notifications (“observed”) and predictions from the final model with a baseline sine wave, season‐specific IAV variables, IBV, RSV and rhinovirus (“predicted”)

However, the number of IAV detections was significantly associated with STSS notifications in the same week (*P* = 0.007, Table S2). When season‐specific IAV variables were used, for three seasons the IAV *P*‐value was below 0.1, and beta coefficients differed notably by season. The model with season‐specific IAV variables was selected as final model (Table 1). Figure 4 shows the observed and predicted (by the final model) number of weekly STSS notifications.

**Table 1:** Final model predicting weekly number of STSS notifications and the IAV‐attributable number of STSS cases according to the model

Predictor	Beta coefficient	95% CI	*P*‐Value	IAV‐attributable number/all STSS notifications (%)
Sine	0.8444	(0.5118‐1.177)	<0.001	
Cosine	0.3165	(−0.0852‐0.7182)	0.123	
IAV 2010‐2011	0.0335	(−0.0031‐0.07)	0.072	29/71 (41%)
IAV 2011‐2012	0.0087	(−0.017‐0.0344)	0.508	7/68 (10%)
IAV 2012‐2013	0.0196	(0.0011‐0.038)	0.038	47/119 (39%)
IAV 2013‐2014	–	–	–	0/50 (0%)
IAV 2014‐2015	0.0038	(−0.0032‐0.0109)	0.287	13/74 (18%)
IAV 2015‐2016	0.0010	(−0.0061‐0.0081)	0.785	3/64 (5%)
IAV 2016‐2017	0.0109	(0.0006‐0.0211)	0.037	48/118 (41%)
IAV 2017‐2018	0.0092	(−0.0096‐0.0279)	0.339	28/83 (34%)
IBV	–	–	–	
RSV	–	–	–	
Rhinovirus	–	–	–	
Intercept	1.4393	(0.853‐2.0256)	<0.001	

Note

Disregarded negative association (non‐significant).

Abbreviations: CI, confidence interval; IAV, influenza A virus; IBV, influenza B virus; RSV, respiratory syncytial virus; STSS, streptococcal toxic shock syndrome.

Applying the beta coefficients from the final model to the number of IAV detections per season, this would imply a total of 175 STSS cases to have been attributable to IAV during the study period, which is 27% of all 647 STSS cases in this period. Notably, in the three seasons with IAV *P*‐values below 0.1, around 40% of the notified STSS cases was attributed to IAV activity.

## 4 DISCUSSION

A significant association was observed between the number of IAV detections and STSS notifications in the Netherlands, without a delay between the 2 time series. None of the other outcomes, that is necrotising fasciitis, puerperal GAS or GAS cultured from blood, upper or lower respiratory tract specimens, genital tract specimens or pus or wounds, were associated with any of the respiratory virus detections. These results suggest that IAV circulation increases the risk of STSS, the most severe iGAS complication.

Although studies on the interaction between IAV and (i)GAS are scarce, some studies have also reported that influenza‐iGAS coinfection increases iGAS disease severity. For example, in a mouse model of influenza A‐GAS superinfection, Okamoto et al^16^ found that IAV infection 2 days prior to GAS infection drastically increased invasive GAS disease and mortality rates. None of the mice infected with GAS strains from human STSS cases died without IAV infection, but up to 90% of the superinfected mice (IAV + STSS strain) died. The mortality was highest when the interval between IAV and GAS infection was 2‐4 days, which is consistent with the association between IAV and STSS within the same week observed in the current study. Indeed, in the data linkage study of an iGAS increase by Zakikhany et al,^7^ the majority of patients had been diagnosed with influenza in the 7 days prior to invasive bacterial disease. Both hemagglutinin and neuraminidase genotypes of IAV have been reported to influence bacterial superinfection severity, which supports our finding that the IAV‐STSS association differed per influenza season, since the circulating influenza strains differ by season.^17^, ^18^ Indeed, not all high IAV seasons in our study period coincided with increased STSS notifications (Table 1), most likely reflecting such an IAV strain dependency.

A time‐series regression similar to ours found influenza B rather than influenza A to be associated with iGAS detections in Montréal during 1996‐2008.^11^ Therefore, it was surprising that we did not find an association with influenza B. This might be due to different choices in model construction; the Montréal study incorporated six sine/cosine pairs, which either may be a better approximation of the baseline or may compete away a larger part of the seasonal influenza A association compared to our model. Our model, with one sine/cosine pair, also potentially overestimates the baseline at the expense of IAV. This is illustrated by season 2013‐2014, when the number of STSS notifications fell below the baseline sine wave of our model, resulting in a negative beta coefficient for IAV (see Table 1 and Figure 4). Indeed, 2013‐2014 was a very mild influenza season in the Netherlands,^19^ illustrating that the baseline sine wave of GAS disease might not be a natural fluctuation entirely independent of IAV activity. When removing the baseline sine wave from our final model, beta coefficients for IAV increased (reflecting a stronger association) but no additional respiratory viruses or IAV seasons showed a significant association with STSS. Further, developments in influenza diagnostic tests affected the influenza time series within the Montréal study period according to the authors and may also explain some differences between the period 1996‐2008 and the current study period.^20^

The current study has important limitations. First of all, it is an ecological study; only associations in time on a population level were estimated which has the risk of finding spurious associations as well as missing actual associations. Further, for the respiratory viruses, the population coverage of the VWR surveillance system is not precisely known and may vary over time as reporting by laboratories is voluntary and laboratories can merge. Other unknown surveillance artefacts regarding potential variations over time in laboratory test request behaviour, and technical testing practices may also play a role. For the GAS cultures, the coverage of the ISIS‐AR surveillance system increased during the study period. Further, the notification criteria for puerperal GAS changed in 2016, which is reflected in the increased number of notifications. The secular trend added to the puerperal GAS and ISIS‐AR models crudely adjusts for these increases but may have left residual surveillance artefacts in the data. Also, the formal iGAS surveillance of notifiable disease presentations is partially limited in the Netherlands as national surveillance data on GAS pneumonia are absent (because not notifiable), while it is strongly associated with STSS.^21^ IAV infection is known to predispose for bacterial pneumonia.^22^ However, we did not observe an association between respiratory viruses and GAS cultures from lower respiratory tract specimens, which might be a proxy measure for GAS pneumonia. Strengths of this study include the multiple GAS outcomes (three disease presentations and five culture specimen types), which enabled the distinction between diseases with differing severity and of different organ systems. In addition, the inclusion of four respiratory viruses enabled the detection of a specific association with IAV, rather than a general co‐seasonality of iGAS disease and respiratory virus infections in winter and early spring.

Our study supports the notion that iGAS burden may be reduced by influenza prevention. Indeed, a case‐control study among US military found a strong protective effect of influenza vaccination on GAS disease.^23^ Further research is needed to estimate the STSS disease burden preventable by seasonal influenza vaccination, such as a case‐control study to ascertain the influenza (vaccination) status of STSS cases and (matched) controls. Such a study would ideally include an elderly population, as people of age 60 and older are eligible for influenza vaccination in the Netherlands and are also at highest risk of STSS (Figure S1).

Our finding of a specific association between IAV and STSS seasonality and not other (i)GAS manifestations should be interpreted with caution as it is an ecological association and needs further confirmation in future studies. However, we believe our study adds a population perspective to the evidence from experimental studies and clinical case reports that influenza significantly impacts iGAS severity. Replication of our study in other settings or systematic influenza testing of iGAS patients may provide more insight into the burden attributable to influenza‐iGAS superinfection.
