# Supplementary figures and images for: Associations between common respiratory viruses and invasive group A streptococcal infection: A time‐series analysis
Source: Influenza Other Respir Viruses. 2019 Jun 25;13(5):453–8. doi: 10.1111/irv.12658 (PMC6692538; doi:10.1111/irv.12658)

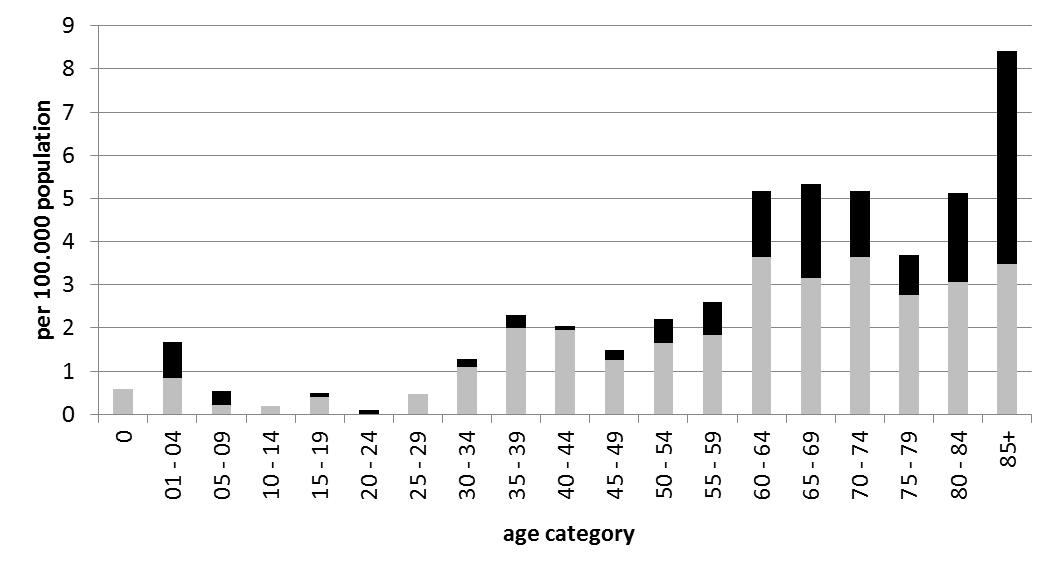

Supplement: Supplementary file 1 [file IRV-13-453-s001.tif]

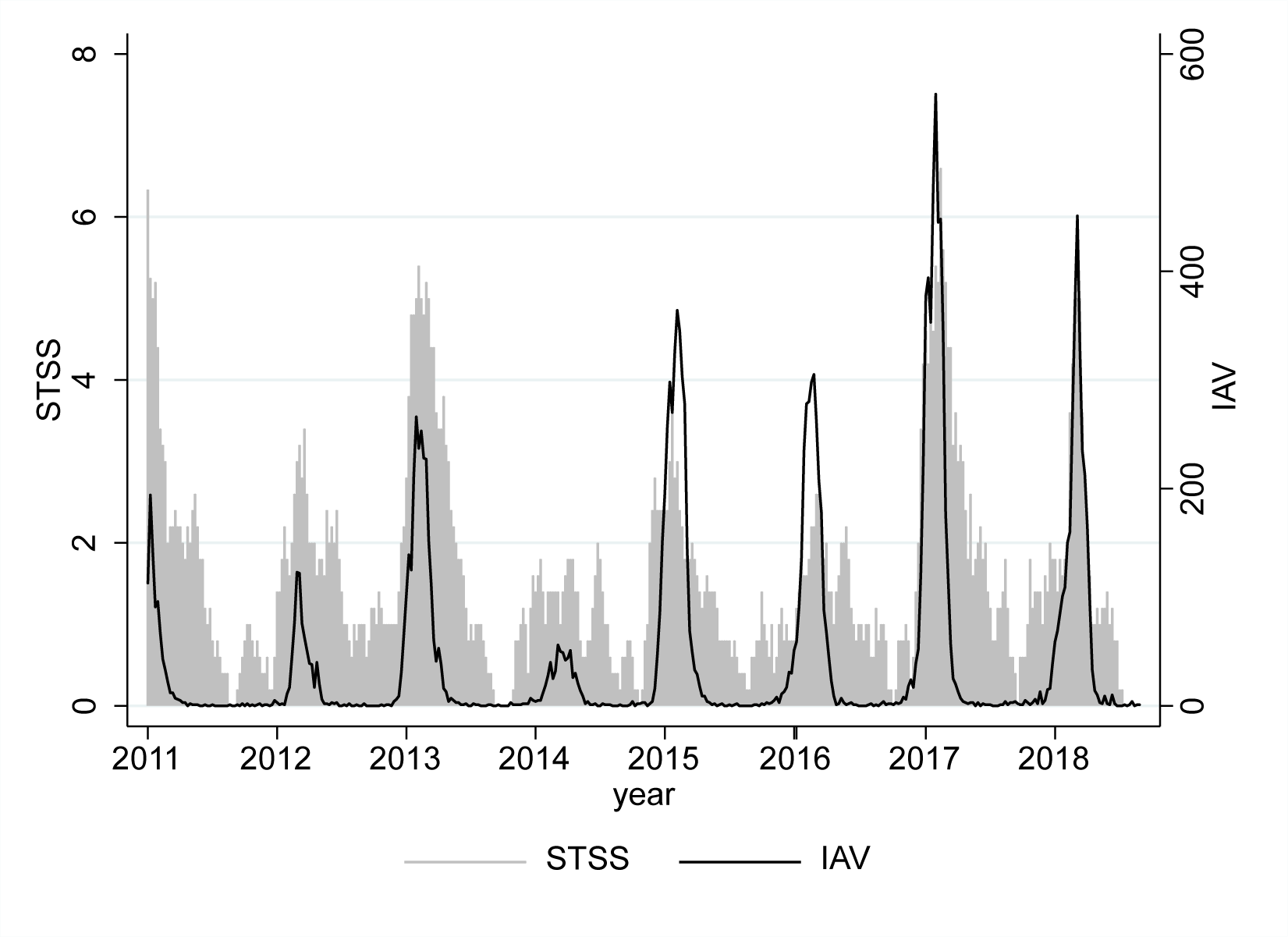

Supplement: Supplementary file 2 [file IRV-13-453-s002.tif]
