# Supplementary material for: Associations between common respiratory viruses and invasive group A streptococcal infection: A time‐series analysis
Source: Influenza Other Respir Viruses. 2019 Jun 25;13(5):453–8. doi: 10.1111/irv.12658 (PMC6692538; doi:10.1111/irv.12658)
Supplement: Supplementary file 3 [file IRV-13-453-s003.docx]

Supplementary Table 1. Number of respiratory virus detections, iGAS disease notifications and GAS cultures, 2008- week 26 2018.

| year | 2008 | 2009 | 2010 | 2011 | 2012 | 2013 | 2014 | 2015 | 2016 | 2017 | 2018 (first 26 weeks) |
| --- | --- | --- | --- | --- | --- | --- | --- | --- | --- | --- | --- |
| Respiratory virus detections (VWR) | | | | | | | | | | | |
| IAV | 234 | 7419 | 158 | 872 | 891 | 2333 | 899 | 3195 | 3117 | 3968 | 2950 |
| IBV | 203 | 120 | 63 | 466 | 64 | 981 | 47 | 698 | 1351 | 397 | 5973 |
| RSV | 2331 | 2030 | 2778 | 2466 | 2043 | 1864 | 1456 | 1870 | 2085 | 1509 | 1409 |
| rhinovirus | 899 | 1994 | 1906 | 1987 | 1780 | 2049 | 2194 | 2410 | 2589 | 2706 | 1603 |
| iGAS disease notifications (OSIRIS) | | | | | | | | | | | |
| STSS | *ni* | *ni* | *ni* | 84 | 84 | 103 | 64 | 66 | 70 | 120 | 56 |
| Necrotising fasciitis | *ni* | *ni* | *ni* | 50 | 53 | 66 | 54 | 53 | 57 | 72 | 25 |
| Puerperal GAS | *ni* | *ni* | *ni* | 17 | 34 | 28 | 27 | 46 | 55 | 92 | 50 |
| GAS cultures (ISIS-AR) | | | | | | | | | | | |
| Blood | 194 | 258 | 234 | 274 | 262 | 377 | 274 | 286 | 281 | 350 | *ni* |
| Upper respiratory tract materials | 447 | 565 | 574 | 575 | 523 | 517 | 413 | 443 | 476 | 567 | *ni* |
| Lower respiratory tract materials | 110 | 159 | 135 | 133 | 120 | 179 | 137 | 170 | 169 | 185 | *ni* |
| Genital tract materials | 711 | 950 | 1041 | 1125 | 1027 | 1254 | 1073 | 1234 | 1190 | 1479 | *ni* |
| Pus/wound | 1780 | 2259 | 2534 | 2406 | 2252 | 2906 | 2346 | 2572 | 2641 | 3190 | *ni* |

Ni: not included in the models. STSS: streptococcal toxic shock syndrome.
