# Supplementary material for: Associations between common respiratory viruses and invasive group A streptococcal infection: A time‐series analysis
Source: Influenza Other Respir Viruses. 2019 Jun 25;13(5):453–8. doi: 10.1111/irv.12658 (PMC6692538; doi:10.1111/irv.12658)
Supplement: Supplementary file 4 [file IRV-13-453-s004.docx]

Supplementary Table 2. Model predicting weekly number of STSS notifications with one IAV variable over seasons, and the IAV-attributable number of STSS cases according to the model

| Predictor | Beta coefficient | 95% CI | P-value | IAV-attributable number / all STSS notifications (%) |
| --- | --- | --- | --- | --- |
| Sine | 0.7548 | (0.4527-1.0569) | <0.001 |  |
| Cosine | 0.1879 | (-0.1928-0.5687) | 0.333 |  |
| IAV | 0.0084 | (0.0023-0.0145) | 0.007 | 153/647 (24%) |
| IBV | -- | -- | -- |  |
| RSV | -- | -- | -- |  |
| Rhinovirus | -- | -- | -- |  |
| intercept | 1.2773 | (0.7222-1.8324) | <0.001 |  |

. -- : disregarded negative association. CI: confidence interval; IAV: influenza A virus; IBV: influenza B virus; RSV: respiratory syncytial virus; STSS: streptococcal toxic shock syndrome.
